# Selections

**Published:** 1895-04

**Authors:** 


### Selections.

### SULPHURIC ACID FOR OPENING ROOT-CANALS.

To illustrate the method, let us suppose we have a superior molar tooth, from which the pulp tissue has been removed; the palatine root, being large, can be prepared by any method you may choose; place the solution in that portion of the cavity near the buccal roots and seal it in the tooth for twenty-four to forty-eight hours; then, upon the removal of the stopping, wash out the cavity with a dash of water from the syringe; upon drying the cavity you will find it white and clean, with two dark spots in the vicinity of the buccal roots, showing where the canals can be found. Now we try to enter the canal with the nerve-bristle: we find no opening. To make sure we are not being deceived by a constriction, we take a bud drill and follow these stains a short distance; if we find no opening, or a very minute opening too small for the bristle, we will feel justified in saying they need no further treatment. But if with our exploring instrument we find a canal, we will carry the acid to the canal by dipping the instrument in the solution, or by means of the pliers, or, better still, the latest pattern of the Dunn syringe, place a drop of the agent in the chamber, and with a No. 5 Donaldson canal cleanser pump the acid into the canal; the acid will soften the walls of the canal sufficiently to allow the broach to cut its way into the root; the acid will also thoroughly sterilize the canal and everything in it. No germ or spore can live in the presence of $H_2SO_4$ in the strength employed,—twenty to fifty per cent. aqueous solution. The broach may scarcely enter the canal at first, but if you are persistent it will be but a few minutes till the instrument will go quite a distance into the canal until you reach the end of the root, where a much stronger resistance will be met with.

The thickened cementum at this point seeming to offer a greater resistance to the agents used, the canal can then be enlarged by using larger broaches, or if the root be straight the "Gates-Glidden" drill will follow the canal just made; it is more than likely that the apical foramen has not yet been opened; this can be accomplished, if desired, by drilling or by placing a small thread of cotton saturated with the acid solution in the end of the root and leaving it there over night, using the broach and acid at the next sitting; after one or two trials you can readily see how crooked or obstructed canals may be opened in a few minutes, and the canal will be in condition for immediate root-filling. It must be borne in mind that the rubber dam should always be in place before the operation is begun.—J. R. CALLAHAN, D.D.S.

